# 基于铁死亡相关基因构建肺腺癌预后模型及验证

**DOI:** 10.3779/j.issn.1009-3419.2025.102.04

**Published:** 2025-01-20

**Authors:** Zhanrui ZHANG, Wenhao ZHAO, Zixuan HU, Chen DING, Hua HUANG, Guowei LIANG, Hongyu LIU, Jun CHEN

**Affiliations:** ^1^832003 石河子，石河子大学医学院第一附属医院胸外科; ^1^Department of Thoracic Surgery, First Affiliated Hospital, School of Medicine, Shihezi University, Shihezi 832003, China; ^2^300052 天津，天津医科大学总医院肺部肿瘤外科; ^2^Department of Lung Cancer Surgery; ^3^天津市肺癌研究所，天津市肺癌转移与肿瘤微环境重点实验室; ^3^Tianjin Key Laboratory of Lung Cancer Metastasis and Tumor Microenvironment, Tianjin Lung Cancer Institute, Tianjin Medical University General Hospital, Tianjin 300052, China

**Keywords:** 肺肿瘤, 铁死亡, 预后模型, Lung neoplasms, Ferroptosis, Prognostic model

## Abstract

**背景与目的:**

铁死亡相关基因在调控细胞内铁稳态和脂质过氧化中发挥关键作用，并且参与调控肿瘤的生长与耐药。铁死亡相关基因在肿瘤组织中的表达可用来预测患者未来的生存时间，帮助医生和患者预测疾病未来的进展。基于癌症基因组图谱（The Cancer Genome Atlas, TCGA）数据库中肺腺癌（lung adenocarcinoma, LUAD）患者的测序数据，本研究筛选出参与铁死亡调控的基因，构建了预后模型，并评估了该模型的预测效果。

**方法:**

由GeneCards数据库提供1467个铁死亡相关基因。TCGA数据库提供了541个LUAD患者的mRNA表达矩阵以及临床数据，提取所有影响铁死亡的基因的表达数据，并利用R软件筛选出癌组织与癌旁组织差异表达的影响铁死亡的基因。对这些基因进行生存分析，以筛选出与预后相关的基因。接着，采用*LASSO*回归模型构建由影响铁死亡的基因组成的预后模型。对所有LUAD患者样本进行评分，并根据中位数分为高风险组和低风险组。随后，绘制受试者操作特征（receiver operating characteristic, ROC）曲线并计算曲线下面积（area under the curve, AUC），由*Kaplan-Meier*生存曲线检验模型的性能，以及在外部数据集中验证。最后，利用单因素*Cox*分析确定模型是否有意义，多因素*Cox*分析探讨模型的独立预后价值以及临床相关性。

**结果:**

通过生存分析，初步筛选出121个与预后相关的铁死亡基因。在此基础上，利用*LASSO*回归构建了一个由12个影响铁死亡的基因（*ALG3*、*C1QTNF6*、*CCT6A*、*GLS2*、*KRT6A*、*LDHA*、*NUPR1*、*OGFRP1*、*PCSK9*、*TRIM6*、*IGF2BP1*和*MIR31HG*）组成的预后模型用以预测LUAD患者的生存时间。结果表明，高风险组患者的生存时间明显少于低风险组（*P*<0.001），并且在训练集（1年AUC=0.721）和外部验证集（1年AUC=0.768）中均展现出不错的预测结果。患者的风险得分在单因素*Cox*分析和多因素*Cox*分析中与LUAD患者的预后显著相关（*P*<0.001），提示该评分是LUAD患者的重要预后因素。

**结论:**

本研究成功构建了一个由12个影响铁死亡的基因组成的LUAD风险评分模型。未来，该模型有望与肿瘤原发灶-淋巴结-转移分期系统联合应用于LUAD患者的预后预测中。

## Abstract (en)

**Background and objective:**

Ferroptosis-related genes play a crucial role in regulating intracellular iron homeostasis and lipid peroxidation, and they are involved in the regulation of tumor growth and drug resistance. The expression of ferroptosis-related genes in tumor tissues can be used to predict patients' future survival times, aiding doctors and patients in anticipating disease progression. Based on the sequencing data of lung adenocarcinoma (LUAD) patients from The Cancer Genome Atlas (TCGA) database, this study identified genes involved in the regulation of ferroptosis, constructed a prognostic model, and evaluated the predictive performance of the model.

**Methods:**

A total of 1467 ferroptosis-related genes were obtained from the GeneCards database. Gene expression profiles and clinical data from 541 LUAD patients were collected from the TCGA database. The expression data of all ferroptosis-related genes were extracted, and differentially expressed genes were identified using R software. Survival analysis was performed on these genes to screen for those with prognostic value. Subsequently, a prognostic risk scoring model for ferroptosis-related genes was constructed using *LASSO* regression model. Each LUAD patient sample was scored, and the patients were divided into high-risk and low-risk groups based on the median score. Receiver operating characteristic (ROC) curves were plotted, and the area under the curve (AUC) was calculated. *Kaplan-Meier* survival curves were generated to assess model performance, followed by validation in an external dataset. Finally, univariate and multivariate *Cox* regression analyses were conducted to evaluate the independent prognostic value and clinical relevance of the model.

**Results:**

Through survival analysis, 121 ferroptosis-related genes associated with prognosis were initially identified. Based on this, a LUAD prognostic risk scoring model was constructed using 12 ferroptosis-related genes (*ALG3*, *C1QTNF6*, *CCT6A*, *GLS2*, *KRT6A*, *LDHA*, *NUPR1*, *OGFRP1*, *PCSK9*, *TRIM6*, *IGF2BP1* and *MIR31HG*). The results indicated that patients in the high-risk group had significantly shorter survival time than those in the low-risk group (*P*<0.001), and the model demonstrated good predictive performance in both the training set (1-yr AUC=0.721) and the external validation set (1-yr AUC=0.768). Risk scores were significantly associated with the prognosis of LUAD patients in both univariate and multivariate *Cox* regression analyses (*P*<0.001), suggesting that this score is an important prognostic factor for LUAD patients.

**Conclusion:**

This study successfully established a LUAD risk scoring model composed of 12 ferroptosis-related genes. In the future, this model is expected to be used in conjunction with the tumor-node-metastasis (TNM) staging system for prognostic predictions in LUAD patients.

全球范围内癌症导致的死亡，肺癌位列第一^[1]^。中国肺癌的发病率和死亡率在所有肿瘤中均居于首位^[2]^。根据病理分型，非小细胞肺癌占比80%-85%，其中肺腺癌（lung adenocarcinoma, LUAD）的发病率逐渐上升，尤其是在不吸烟的女性中。肺癌患者的预后通常较差，主要原因是肺癌早期不易发现。在临床上，常用的LUAD预后预测指标包括肿瘤大小、淋巴结转移、组织学特征和肿瘤突变负荷等。然而，由于肿瘤的高度异质性，即使在相同肿瘤原发灶-淋巴结-转移（tumor-node-metastasis, TNM）分期的患者中，疗效和预后也可能存在显著差异。因此，单纯依赖这些指标往往无法准确评估患者的预后，需要探索新的预后标志物，以辅助现有的预测指标，从而更有效地评估LUAD患者的预后，为患者的个体化治疗提供更有力的支持。

铁死亡是一种新型的细胞死亡方式，主要由Fe²^+^或脂氧合酶的作用引发，导致以羟基为代表的活性氧引起脂质过氧化，从而损害细胞膜结构^[3]^。这一过程与传统的细胞死亡方式（如凋亡、坏死和自噬）有显著不同。肿瘤细胞的代谢水平明显高于正常细胞，使其产生更多的活性氧，从而对铁死亡更加敏感^[4]^。许多肿瘤细胞通过抵抗铁死亡在高氧化压力的环境中生存^[5]^。通过促进肿瘤细胞的铁死亡，可以有效抑制其增殖和存活。多种化疗药物、中药和放疗均可通过诱导铁死亡来增强治疗效果，铁死亡这种独特的细胞死亡机制有可能被用于解决临床耐药问题^[6⇓-8]^。所以铁死亡在肿瘤中发挥重要作用，研究铁死亡在肿瘤中的具体机制可能对肿瘤治疗提供新思路。

多个基因构建的预后风险模型在预测各种肿瘤预后方面得到了广泛关注。这些模型在卵巢癌、结肠癌等多种癌症中表现出色^[9⇓-11]^。本研究利用生物信息学技术，通过深入分析癌症基因组图谱（The Cancer Genome Atlas, TCGA）数据库中LUAD患者的基因表达数据，筛选出在肿瘤与正常组织中差异表达且与LUAD预后相关的铁死亡基因。识别出的这些铁死亡相关的基因在LUAD预后中可能具有重要价值。我们采用*LASSO*回归分析成功构建铁死亡相关基因组成的LUAD预后风险评分模型并评估该模型的预测性能，使用其他数据库来验证模型在不同患者群体中的适用性。我们确定LUAD患者的危险得分是重要的预后指标，这提示筛选出的基因在肿瘤中发挥重要作用，为探究这些基因的功能，我们通过通路富集探究这些基因的功能。

## 1 资料与方法

### 1.1 数据获取

在GeneCards数据库（https://www.genecards.org/）中检索“Ferroptosis”，共获得1467个铁死亡相关基因。2024年9月13日，从TCGA数据库下载了541例LUAD样本及59例配对的正常肺组织样本的RNA测序数据。同时，收集患者的临床信息，包括性别、年龄、临床分期、生存状态及生存时间。此外，来自基因表达综合数据库（Gene Expression Omnibus, GEO）的数据集GSE31210用来验证模型。本研究还使用了天津医科大学总医院肺部肿瘤外科31例LUAD患者临床数据与RNA测序进行验证。

### 1.2 筛选和可视化差异表达的铁死亡基因

本研究利用R-4.4.1软件中的“limma”包，筛选LUAD组织与癌旁组织间的差异表达铁死亡相关基因，设定标准为伪发现率（false discovery rate, FDR）<0.05和|log2(Fold Change)|>1，以确保基因表达差异显著。筛选后，通过生存分析评估基因与总生存期（overall survival, OS）的关系，设定阈值为*P*<0.05，以确定具有预后价值的差异表达铁死亡基因。使用“pheatmap”包绘制热图，展示基因在LUAD组织与癌旁组织中的表达模式，同时利用“venn”包绘制韦恩图。蛋白互作网络由STRING数据库生成。

### 1.3 构建预后风险模型

将LUAD患者的临床信息与铁死亡相关基因的RNA-Seq数据合并，去除无生存时间记录的患者后，共纳入503例LUAD患者。在训练集中筛选出121个铁死亡相关基因，这些基因具有预后价值，利用*LASSO*回归构建预后模型，并绘制森林图和热图。风险评分的计算公式为：RiskScore=基因1表达量×Coef1+基因2表达量×Coef2+…+基因*n*表达量×Coef*n*（其中，Coef为基因在*LASSO*回归中的回归系数，*n*为*LASSO*回归分析得到的基因数目）。通过模型为每位患者赋予1个风险得分，按照得分的中位数将患者分为低风险组和高风险组。

### 1.4 评价预后风险模型

本研究利用R软件的“survminer”包绘制了*Kaplan-Meier*生存曲线，比较高风险组和低风险组的OS，直观展示风险评分对患者生存的影响。同时，我们加载“survival”包，为了直观展示不同风险组之间的生存差异，绘制评分分布曲线及患者生存情况分布图。应用“timeROC”包，为评估模型的预测能力，绘制受试者工作特征（reciever operating characteristic, ROC）曲线，计算样本OS在1、2和3年的曲线下面积（area under the curve, AUC）用来评价预测效果。使用“Rtsne”包进行主成分分析（principal component analysis, PCA）和t-分布随机邻域嵌入（t-distributed stochastic neighbor embedding, t-SNE）分析，对数据进行降维处理，以显示模型对高、低风险组的区分能力。

### 1.5 验证预后风险模型

为了防止构建的预后模型过拟合，我们使用GO数据库（GSE31210）作为外部数据集进行验证，按照同样的标准进行打分与分组。为评估预后风险模型的预测性能，我们绘制了*Kaplan-Meier*生存曲线，以展示不同风险评分组别患者的生存差异。同时为评估模型的预测能力，绘制ROC曲线。风险评分分布曲线与生存情况分布图则揭示了不同风险组别患者的生存状态与风险评分之间的关系。这些可视化结果用以验证模型的有效性。

### 1.6 模型独立预后分析

对风险评分进行单因素、多因素*Cox*分析，以评估其独立预后价值。如果在这两种分析中均显示出显著差异，说明风险评分是影响预后的独立危险因素。

### 1.7 预后风险得分与临床指标的相关性

通过TCGA构建的预后模型对真实临床病例按照同样的标准进行打分与分组，统计高、低风险组患者的临床分期、TNM分期、淋巴结转移、是否侵及胸膜等与预后相关的临床指标。分析预后风险得分与临床指标的相关性。

### 1.8 通路富集分析

利用R软件的“clusterProfiler”和“enrichplot”等R包，对高风险组与低风险组之间的差异基因进行基因本体功能（Gene Ontology, GO）富集分析和京都基因与基因组百科全书（Kyoto Encyclopedia of Genes and Genomes pathway, KEGG）通路分析及可视化，以探讨构建预后模型的基因的潜在分子机制。

### 1.9 统计学分析

本研究为生信在线数据库分析，所有统计分析均使用R 4.4.1进行。通过单因素*Cox*回归分析*P*<0.05被认为具有统计学意义确定与预后相关的基因，按照FDR<0.05和|log2(Fold Change)|>1筛选肿瘤组织与癌旁组织差异表达的基因。使用*Kaplan-Meier*曲线研究铁死亡相关的风险评分或基因表达与OS的关系。*P*<0.05为有统计学差异。

## 2 结果

### 2.1 预后相关铁死亡基因的筛选

本研究从GeneCards数据库获得了1467个铁死亡相关基因，对TCGA数据库541个LUAD患者样本和59个癌旁组织样本的mRNA表达矩阵进行分析。首先，使用FDR<0.05和|log2(Fold Change)|>1的标准对LUAD组织和正常组织的1467个铁死亡相关基因的mRNA表达数据进行差异分析，识别出了356个差异表达的基因，其中250个上调，106个下调。接着，进行单因素*Cox*分析，得到402个影响预后的基因（*P*<0.05）。随后，将356个差异表达基因与402个预后相关基因交集，最终得到121个目标基因。目标基因的可视化结果如图1A和图1B所示，图1C展示了它们的蛋白互作关系。

**图1:**
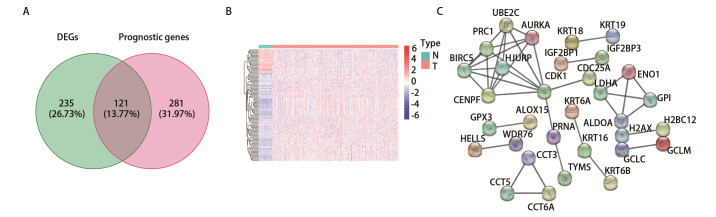
肺腺癌中与预后相关的铁死亡基因筛选。A：肺腺癌差异表达的铁死亡基因与肺腺癌预后相关的铁死亡基因的韦恩图；B：交集基因的热图；C：交集基因的蛋白互作网络。

### 2.2 预后模型的构建

对121个与预后相关的差异表达铁死亡基因进行*LASSO*回归分析，构建出一个由12个基因组成的预后模型。其中，*GLS2*和*NUPR1*的风险比（hazard ratio, HR）<1，提示其低表达与高风险相关；而*ALG3*、*C1QTNF6*、*CCT6A*、*KRT6A*、*LDHA*、*OGFRP1*、*PCSK9*、*TRIM6*、*IGF2BP1*和*MIR31HG*的HR>1，表明其高表达与高风险相关。根据每个基因的表达量与风险系数对每位LUAD患者打分，模型公式为：（0.032*ALG3表达量）+（0.127*C1QTNF6表达量）+（0.004*CCT6A表达量）+（-0.042*GLS2表达量）+（0.028*KRT6A表达量）+（0.204*LDHA表达量）+（-0.054*NUPR1表达量）+（0.049*OGFRP1表达量）+（0.006*PCSK9表达量）+（0.010*TRIM6表达量）+（0.059*IGF2BP1表达量）+（0.036*MIR31HG表达量）。绘制这12个基因的热图（图2A）、树状图（图2B）、相关性网络（图2C）和生存分析（图2D-2O）。对这121个基因进行进行GO功能及KEGG通路富集分析。KEGG信号通路主要富集在铁死亡，同时还富集到金黄色葡萄球菌感染和糖酵解/糖异生等其他通路（补充图1A， http://www.lungca.org/files/2024s207-s1.pdf）。GO通路富集分析进一步揭示了这些差异基因在细胞骨架组织、细胞周期检查点信号传导以及多种生物过程中的作用（补充图1B-1C， http://www.lungca.org/files/2024s207-s1.pdf）。

**图2:**
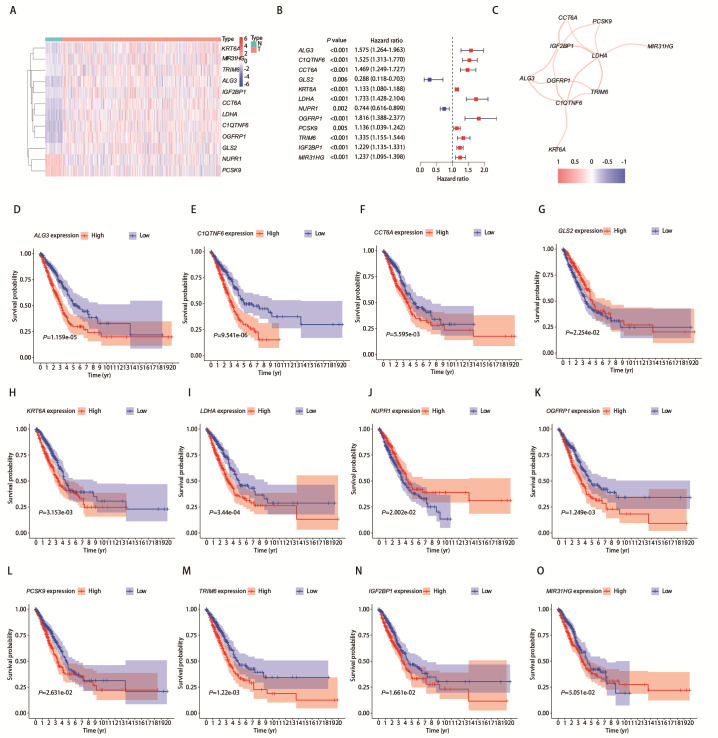
LASSO回归筛选出12个与患者预后高度相关的建模基因。A：建模基因表达热图；B：建模基因单因素Cox分析的森林图；C：建模基因的相关性网络；D-O：12个建模基因的生存分析。

### 2.3 风险模型性能评价

根据12个铁死亡相关基因的表达量及其回归系数，计算每个LUAD样本的风险评分，并根据风险评分的中位数将患者分为高风险组（*n*=251）和低风险组（*n*=252）。*Kaplan-Meier*生存曲线显示，高风险组患者的OS、无进展生存期明显低于低风险组（*P*<0.0001，图3A）。ROC曲线结果如图3B所示，1年AUC为0.721，2年为0.739，3年为0.722。图3C和图3D展示了高、低风险评分的分布曲线及生存情况分布图，结果表明患者风险得分越高预后越差。使用PCA和t-SNE分析对数据进行降维处理，结果显示高、低风险组有效区分（图3E、3F）。

**图3:**
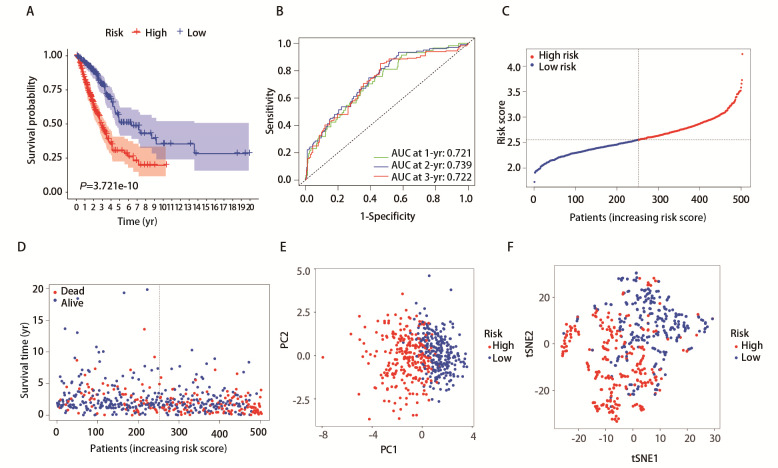
构建的预后模型对TCGA数据库中的样本生存时间与生存状态进行评估。A：Kaplan-Meier生存曲线显示高风险组（红色）与低风险组（蓝色）之间的生存概率差异，P值为3.721e-10；B：时间依赖性ROC曲线，展示了在1、2和3年的AUC值，分别为0.721、0.739和0.722；C：风险评分分布图，患者根据风险评分的增加而排序，高风险组（红色）和低风险组（蓝色）；D：生存时间与状态分布图，红色点表示已死亡患者，蓝色点表示存活患者；E：PCA分析结果，PC1与PC2的散点图显示高风险组（红色）和低风险组（蓝色）的分布情况；F：t-SNE分析结果，显示高风险组（红色）与低风险组（蓝色）的分布，进一步验证了模型对风险组的区分能力。

### 2.4 外部数据集验证模型

在GEO数据库中选取GSE31210数据集，共226个LUAD患者作为外部验证集进行模型验证。根据这12个基因的表达量和系数，对226个LUAD患者进行风险评分，并依据评分进行排序，以中位数为分界线将患者分为高风险组和低风险组。*Kaplan-Meier*生存曲线显示高风险组的生存期显著低于低风险组（*P*<0.0001，图4A）。同时绘制的时间依赖性ROC曲线显示，1年AUC为0.768，2年为0.715，3年为0.662（图4B）。此外，绘制高、低风险评分的分布曲线和生存情况分布图（图4C、4D）。结果表明，高风险组的患者死亡比例明显高于低风险组，提示高风险组患者更易出现不良预后。通过PCA和t-SNE分析对数据进行降维处理，结果显示高、低风险组被有效区分（图4E、4F）。这些结果表明，该模型在外部验证集中仍具有良好的预测性能。

**图4:**
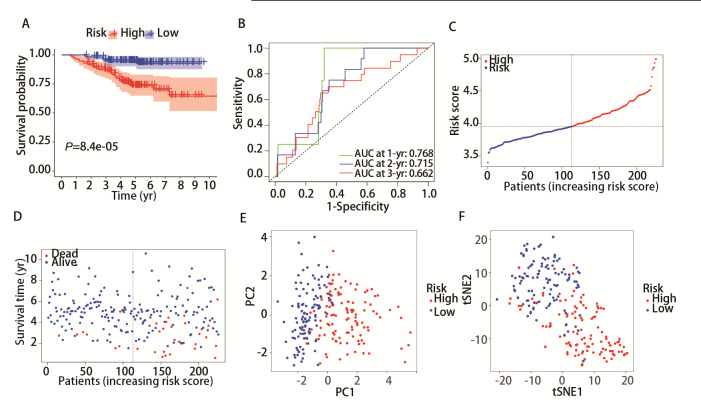
预后风险评分模型在GSE31210中的性能评估。A：GSE31210的Kaplan-Meier生存曲线；B：GSE31210的时间依赖性ROC曲线；C：GSE31210的风险曲线；D：GSE31210的生存状态图；E：GSE31210的PCA分析；F：GSE31210的t-SNE分析。

### 2.5 风险评分具有独立预后价值

本研究纳入LUAD患者的年龄、性别、病理分期和患者风险评分等多个指标通过单因素*Cox*分析探讨对LUAD患者预后的影响。病理分期与风险得分均与预后有关（*P*<0.05，见图5A和5C）。多因素*Cox*分析确认，病理分期与风险得分具有独立预后价值（*P*<0.05，见图5B和5D）。这表明铁死亡相关风险评分在控制其他变量的情况下，依然能够有效预测患者预后。综上所述，单因素*Cox*分析和多因素*Cox*分析显示铁死亡相关风险评分具有独立预后价值，可作为LUAD患者的重要预后预测因子，为临床决策和个性化治疗提供支持。

**图5:**
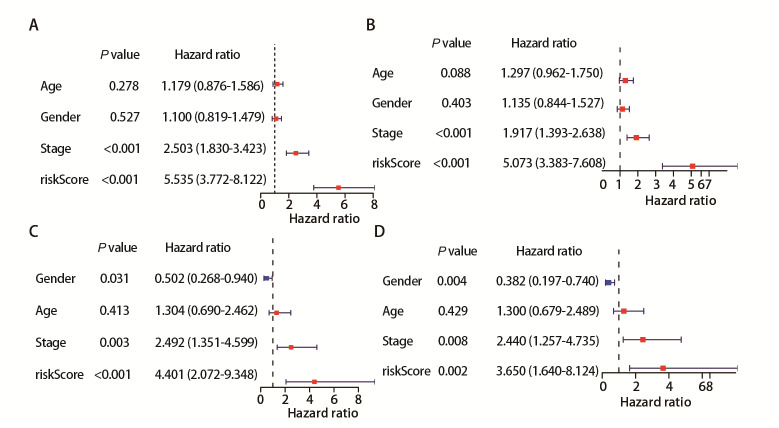
风险得分等多种因素对生存风险的影响。A：单因素Cox分析显示年龄、性别、分期和风险评分对生存风险的影响；B：多因素Cox分析在调整其他变量后，各因素对生存风险的影响；C：GSE31210的单因素Cox分析结果；D：GSE31210的多因素Cox分析结果。

### 2.6 铁死亡预后风险评分与临床指标的相关性

根据12个铁死亡相关基因的表达量及其回归系数，计算31例临床LUAD患者的风险得分，根据得分的中位数将临床样本分为高风险组（*n*=15）与低风险组（*n*=16），统计高、低风险组的临床分期、TNM分期、淋巴结转移、是否侵及胸膜等与预后相关的临床指标。结果显示低风险组患者相较于高风险组患者临床分期更早，较少发生淋巴结转移、侵袭胸膜等影响患者预后的事件（图6A-6F）。这些结果表明该模型对真实病例进行风险打分可以一定程度反映患者的预后。

**图6:**
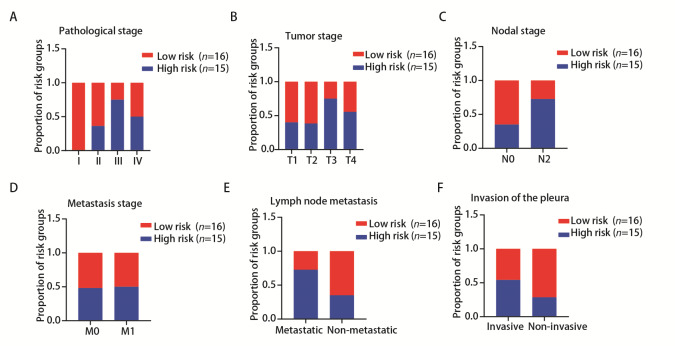
铁死亡预后风险评分与临床指标的相关性。A：预后风险评分高、低风险分组与临床分期的相关性；B-D：预后风险评分高、低风险分组与T、N、M分期的相关性；E：预后风险评分高、低风险分组与淋巴结转移的相关性；F：预后风险评分高、低风险分组与是否侵袭胸膜的相关性。

### 2.7 高、低风险组差异基因的通路富集分析

在TCGA与GSE31210数据集中，以FDR<0.05和|log2(Fold Change)|>1为阈值筛选出高、低风险组的差异表达基因。TCGA数据集中筛选出900个差异基因，其中421个在高风险组低表达，479个在高风险组高表达。在GSE31210数据集中筛选出670个差异基因，其中216个在高风险组低表达，454个在高风险组高表达。对TCGA筛选出的900个差异基因进行KEGG、GO通路富集分析，结果显示，差异基因主要富集在微管结合、细胞骨架马达活性、微管马达活性、糖胺聚糖结合、丝氨酸型内切酶抑制活性等通路中，这些通路在调控细胞骨架、细胞运动和蛋白质的胞内运输等方面发挥重要作用（*P*<0.05，图7A）。KEGG主要富集在了细胞周期、白细胞介素17（interleukin 17, IL-17）信号通路、细胞外基质-受体相互作用等信号通路（*P*<0.05，图7B）。

**图7:**
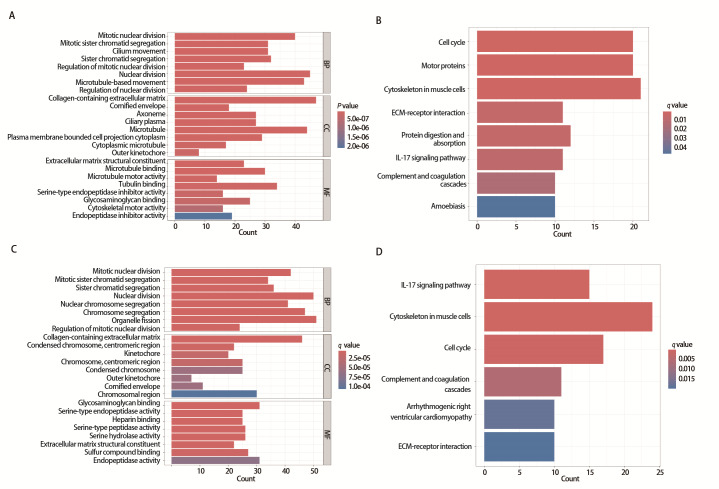
铁死亡预后风险评分相关差异基因的通路富集分析。A：预后风险评分的GO分析；B：预后风险评分的KEGG分析；C：GSE31210的预后风险评分的GO分析；D：GSE31210的预后风险评分的KEGG分析。

对GSE31210数据集中筛选出的670个差异基因进行KEGG、GO通路富集分析，结果显示，差异基因主要富集在糖胺聚糖结合、丝氨酸型内切酶抑制活性、内切酶活性、细胞外基质结构成分等通路中，这些通路在细胞信号传递、细胞间相互作用和蛋白质代谢等方面发挥重要作用（*P*<0.05，图7C）。KEGG主要富集在细胞周期、肌肉细胞中的细胞骨架、IL-17信号通路、细胞外基质-受体相互作用等信号通路（*P*<0.05，图7D）。

## 3 讨论

研究^[12]^表明LUAD通过对铁死亡的抗性，促进了LUAD细胞的生存。多种化疗药物被证实通过促进铁死亡发挥抗肿瘤的作用，促进LUAD的铁死亡也可以降低耐药细胞的耐药性^[13]^。对铁死亡的研究有望为临床治疗提供思路。

本研究通过GeneCards数据库收集铁死亡相关基因，利用来自TCGA的LUAD RNA-seq数据和生存信息，筛选出121个有预后价值并且在肿瘤与正常组织之间差异表达的铁死亡相关基因，通过*LASSO*回归分析筛选出12个关键铁死亡相关基因（*ALG3*、*C1QTNF6*、*CCT6A*、*GLS2*、*KRT6A*、*LDHA*、*NUPR1*、*OGFRP1*、*PCSK9*、*TRIM6*、*IGF2BP1*、*MIR31HG*）构建了LUAD预后风险评分模型。

*ALG3*是一种在内质网中参与糖基化的酶，负责将糖链添加到天冬酰胺上，形成N-糖基化天冬酰胺。*ALG3*在多种肿瘤中高表达，如LUAD、乳腺癌和膀胱癌，且与预后不良有关。敲低*ALG3*可抑制非小细胞肺癌细胞的增殖和迁移，而其上调则增强乳腺癌的放射抗性^[14]^。抑制*ALG3*还会导致N-连接糖基化缺陷，诱导肿瘤细胞发生铁死亡^[15]^。C1QTNF6是C1qTNF相关蛋白家族的一员，已被确认为多种癌症的预后生物标志物^[16]^。抑制C1QTNF6可减弱非小细胞肺癌细胞的增殖和迁移^[17]^。CCT6A参与蛋白质折叠和稳定，其沉默可抑制LUAD细胞的增殖和迁移^[18]^。GLS2是谷氨酸合成的关键酶，作为肿瘤抑制因子，通过促进铁死亡来抑制肿瘤生长^[19]^，缺失GLS2的肝癌细胞对铁死亡抵抗。KRT6A是角蛋白家族的成员，在非小细胞肺癌中上调，促进肿瘤细胞生长和侵袭^[20]^。其与铁死亡的关系较少报道，但在银屑病样皮炎中可抑制GPX4，诱导铁死亡。LDHA催化乳酸与丙酮酸之间的转化，促进肿瘤细胞的无氧代谢，成为抑制肿瘤代谢重编程的重要靶点^[21]^。在结直肠癌中，载脂蛋白L3（apolipoprotein L3, APOL3）可以结合LDHA，促进其降解，从而介导结直肠癌的铁死亡^[22]^。NUPR1是一种转录因子，调节多种基因的表达，与肿瘤细胞的存活和耐药性相关^[23]^。NUPR1被认为是铁死亡的拮抗剂^[24]^。OGFRP1是一种长链非编码RNA，在多种肿瘤中上调，促进肿瘤进展。干扰OGFRP1功能可抑制细胞增殖和迁移，并诱导凋亡^[25]^。在肺癌中OGFRP1可以通过抑制miR-299-3p增强SLC38A1表达来抑制铁死亡从而促进肺癌细胞增殖^[26]^。PCSK9通过与LDLR结合，减少胆固醇摄取，影响结肠癌进展^[27]^。抑制PCSK9可以增加细胞内脂质氢过氧化物的累积，同时破坏p62/Keap1/Nrf2抗氧化轴导致铁死亡^[28]^。TRIM6作为E3泛素连接酶，在肺癌中表达上调，促进肿瘤进展，并抑制谷氨酰胺分解和铁死亡^[29]^。IGF2BP1调节mRNA的稳定性和翻译效率，在多种肿瘤中表达上调，预防铁死亡^[30]^。MIR31HG是一种长链非编码RNA，在多种肿瘤中高表达，调节Wnt/β-catenin和PI3K/Akt信号通路，影响肿瘤生物学特性^[31]^。

上述12个铁死亡相关基因的生存分析和风险评分分布图提示，*GLS2*、*NUPR1*基因低表达和*ALG3*、*C1QTNF6*、*CCT6A*、*KRT6A*、*LDHA*、*OGFRP1*、*PCSK9*、*TRIM6*、*IGF2BP1*、*MIR31HG*基因高表达患者的风险评分高，预后不良。通过风险评分分布及*Kaplan-Meier*生存曲线分析，高风险评分患者的生存时间更短。此外，模型在1、2、3年的AUC值表明其在LUAD预后预测中具备良好的准确性，并在外部验证数据集GSE31210也确认了其预测性能的准确性，显示该模型具有广泛的适用性和可靠性；并且我们使用了真实的临床病例样本的临床指标进行了验证，但由于临床样本数量有限，验证结果可能受到抽样误差和随机波动的影响。但我们将在后续研究中结合更多临床数据进一步完善。KEGG与GO的通路富集分析提示，这些基因的功能集中在调控细胞周期、细胞骨架、IL-17信号等。

本研究构建的预后模型对LUAD患者预后具有一定的预测能力，该模型对患者的风险评价有独立的预后价值同时与临床指标具有相关性，这为临床医生评价患者疾病状况提供参考。研究筛选出的12个与铁死亡相关的基因在多种肿瘤中与肿瘤进展及预后密切相关，其表达变化可能影响肿瘤细胞的生存和转移。深入研究这些基因的分子机制，将揭示铁死亡在肿瘤生物学中的作用，并为肿瘤治疗和肿瘤耐药性的相关研究提供新思路。
